# Accidental Blast Injuries While Making Indigenous Explosive Chemical Mixture: A Report of Two Incidents

**DOI:** 10.7759/cureus.26609

**Published:** 2022-07-06

**Authors:** Devendra Jadav, Sourav Bhowmik, Ashish Saraf, Asharam Gorchiya, Vikas Meshram, Raghvendra S Shekhawat

**Affiliations:** 1 Forensic Medicine and Toxicology, All India Institute of Medical Sciences, Jodhpur, Jodhpur, IND; 2 Forensic Medicine and Toxicology, All India Institute of Medical Sciences, Gorakhpur, Gorakhpur, IND; 3 Forensic Medicine and Toxicology, Ananta Institute of Medical Sciences and Research Centre, Rajasamand, IND

**Keywords:** improvised explosive, chemical accident, sulfur, gandhak, potash, explosive agents

## Abstract

Apart from terrorist and military bombing, accidental blast injuries are the major source of morbidity and mortality in explosion occurrences. In civilian scenarios, it can happen when unskilled individuals handle explosive materials carelessly, often avoiding legal restrictions. We report two incidents of an accidental explosion in which three victims got injured during the mixing of explosive chemicals which are used in an improvised pipe gun to scare away the animals on the farm. In both incidents, the victims were mixing *Gandhak *(sulfur) and *Potash* to make an indigenous fire cracker-type explosive mixture. The victims suffered classical low-order explosion injuries. The chemical reaction between the chemicals, the treatment course of all three victims, and medico-legal implications are also discussed in the article.

## Introduction

An explosion is described as the rapid release of previously contained energy due to the conversion of solid or liquid components into gases, followed by the emission of significant amounts of heat and sound waves [1]. Incidences of explosions in the civilian population apart from the terrorist activity are rare, and mostly accidental [2]. The majority of these incidents occur in industrial facilities [3]. However, in civil areas, the possibility of accidental blasts outside of the industrial facility increases when explosive chemical substances are handled by unskilled personnel. We describe two such instances of an accidental chemical explosion. The incidents occurred while combining explosive chemicals used in an improvised pipe gun to frighten away farm animals. For academic reasons, including publication, written informed consent was obtained from the patients for the use of photographs and medical history.

## Case presentation

Incident 1 

**Figure 1:**
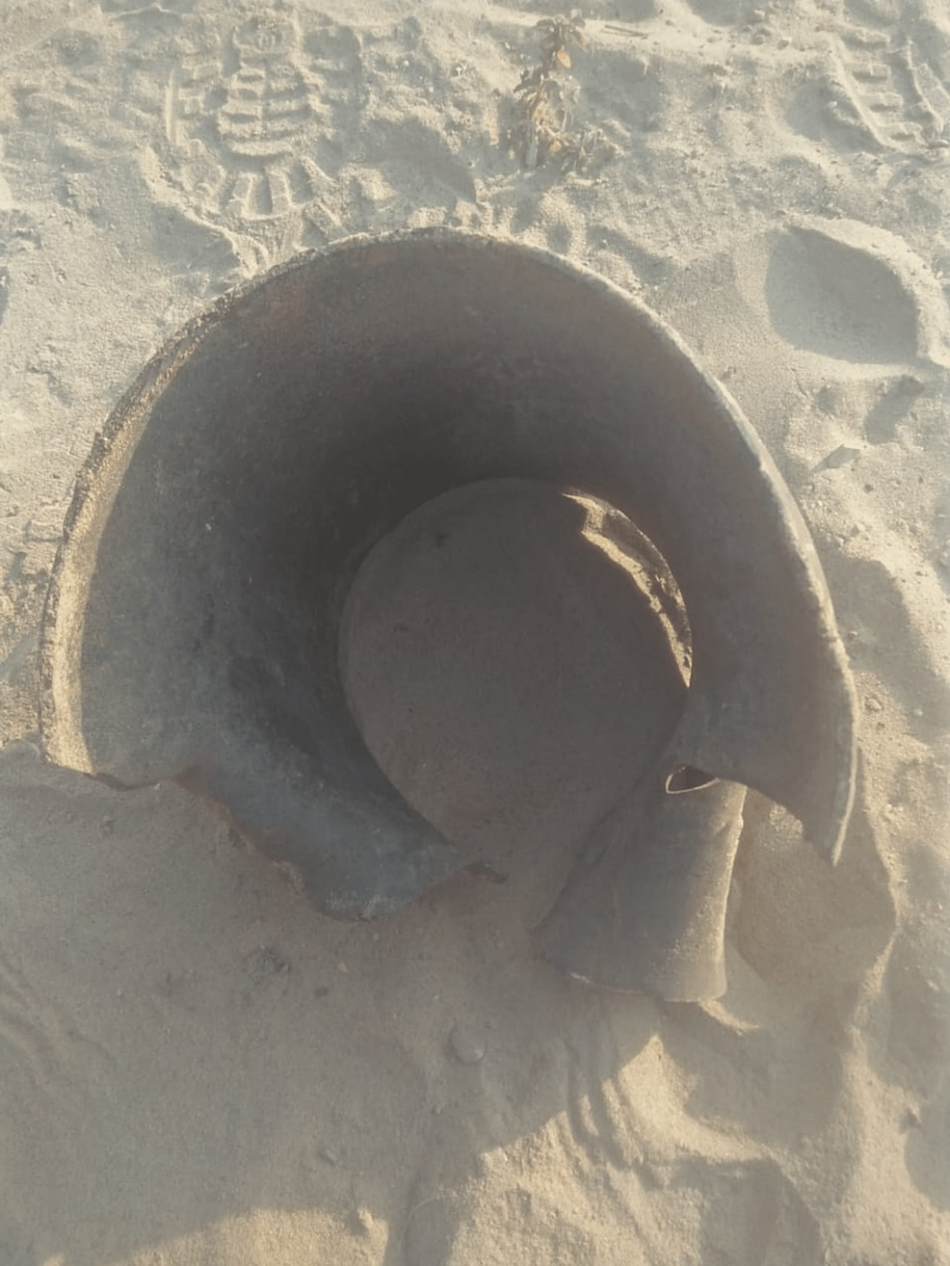
Blasted mortar used for the mixing of chemicals at the scene of the first incident.

Two farmer brothers (22 and 15 years old) were brought to emergency with an alleged history of blast injuries while they were mixing explosive chemicals Gandhak (sulfur) and potash. The event occurred when they were grinding the solid form of around 500 g of both the chemicals using iron mortar and pestle. The blasted mortar is shown in Figure 1.

**Figure 2:**
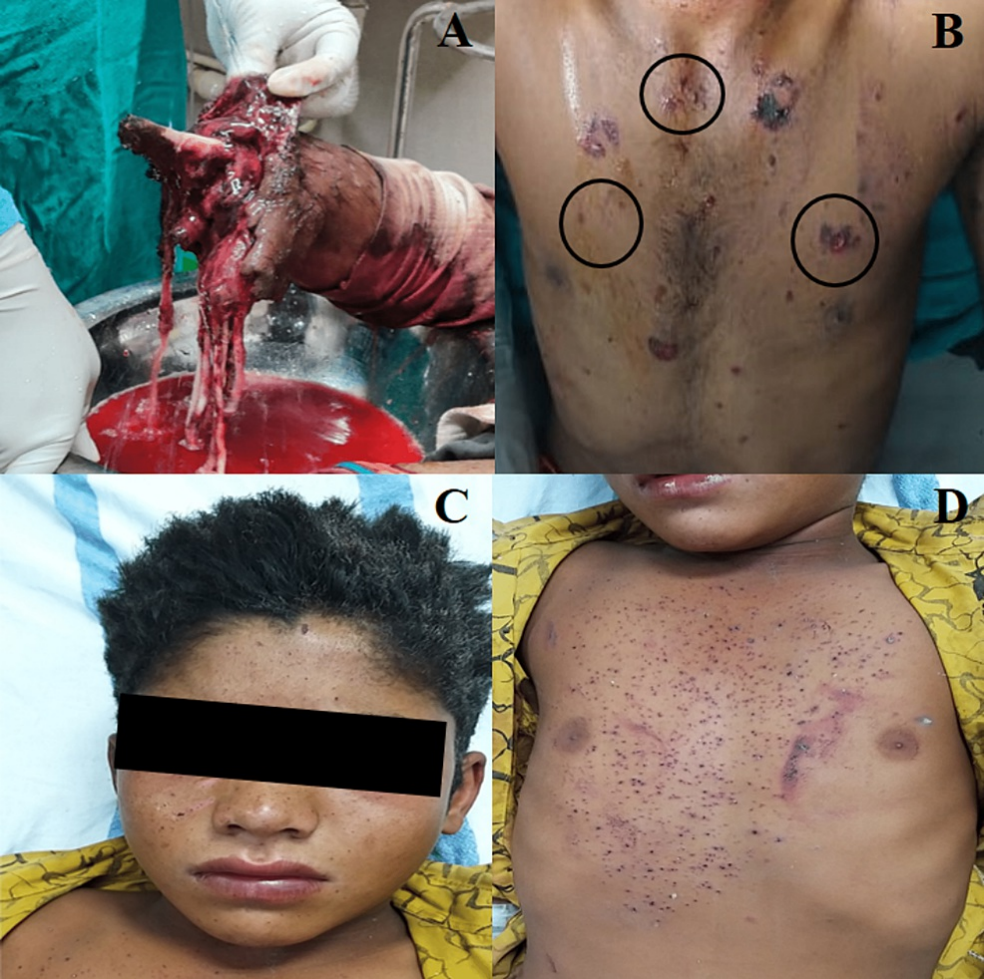
Open grade III-c right forearm below-elbow traumatic amputation (A) along with multiple punctate abrasions, contusions, and lacerations (black circles) over the body (B) of elder brother in incident 1. Powder tattooing over the face along with singeing of scalp hair (C) and extensive tattooing over the chest and abdomen (D) of younger brother in incident 1.

Both of them were unable to open their eyes at presentation. The clothes of both the victims were burnt at places. Blackening, tattooing, and singeing of scalp hair was evident (Figures 2A-2D).

The patient (elder brother) sustained a split laceration of size 4 cm x 0.5 cm x subcutaneous deep over the forehead on the left side. Open grade III-c right forearm below-elbow traumatic amputation (Figure 2A) along with multiple punctate abrasions, contusions, and lacerations were present over the front of the body at places, predominantly over the chest and abdomen (Figure 2B). Laceration of size 5 cm x 4 cm x muscle deep was present over the base of the left thumb. At presentation, the patient (elder brother) was semi-conscious and had pulse rate - 90/min, BP - 136/80 mmHg, respiratory rate - 16/min and SpO_2 _- 99% on room air. Non-contrast computed tomography (NCCT) head revealed fracture of the outer table of frontal bone without any intra-parenchymal injury. Ophthalmic examination revealed multiple foreign bodies in cornea and hyphama of both eyes with vision 6/24 on the right eye and hand movement vision on the left eye. Local debridement with stump closure and stay suture was done on the right forearm on the same day. A split skin graft was taken from the left forearm and was placed on the right forearm on the fifth day. The patient was discharged on the 10th day with advice of follow up visits. At the time of discharge, the patient diagnosed with corneal melting of the right eye, with vision of 6/60 on the right eye was referred to a corneal surgeon for further management.

The second patient (younger brother) presented with extensive “powder tattooing” in the form of multiple black punctate lesions over the face, chest and abdomen (Figures 2C, 2D). Swabs were taken from the sites of blackening and subjected to chemical analysis to know the constituents of the chemicals, which were identified as sulfur and potassium chlorate. At presentation, he had a pulse rate of 92/min, BP- 98/66 mmHg and spo2- 100% on room air. Ophthalmic examination revealed foreign bodies in the corneas with vision 6/12 on the right eye and 6/36 on the left eye. The patient was started on antibiotics, and foreign bodies were removed under general anesthesia on the second day. He was discharged on the third day with vision 6/6 on the right eye and 6/24 on the left eye with the advice of follow up visit.

Incident 2

**Figure 3:**
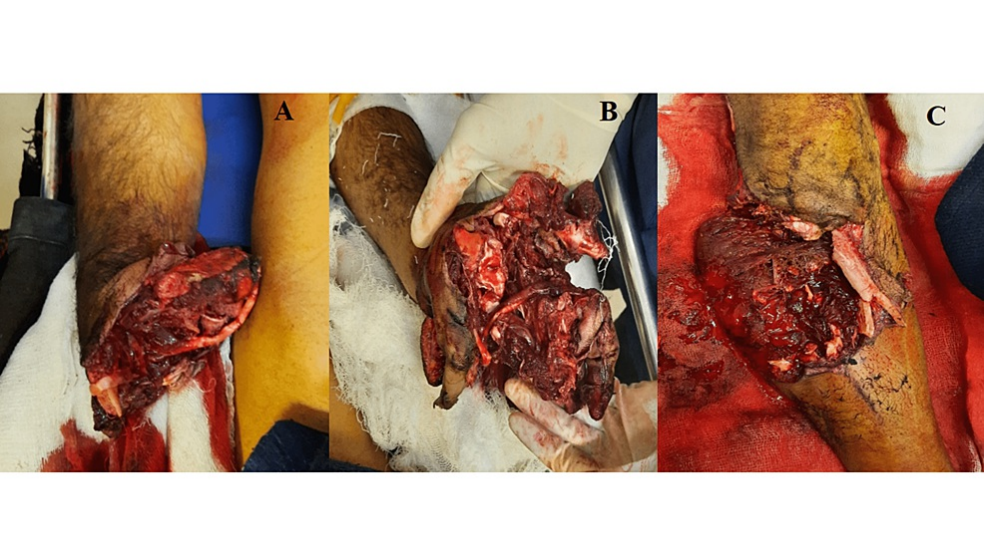
Right (A) and left (B) forearm open grade III-c below elbow traumatic amputation along with open comminuted fracture of the tibia of the right leg (C) of the victim in incident 2.

A 35-year-old farmer was brought to the emergency with an alleged history of explosion injuries sustained while making the explosive chemical mixture of around 500 g of Gandhak and Potash on a piece of paper with iron pestle. The patient was in a semi-conscious state at the time of presentation. The patient had a pulse rate - 106/min, BP - 146/58 mmHg, respiratory rate - 21/min and SpO_2_ - 99% on room air. Blackening, tattooing, and singeing of scalp hair was evident. Swabs were taken from the sites of blackening and subjected to chemical analysis, which were identified as sulfur and potassium chlorate. The victim sustained bilateral forearm open grade III-c below elbow traumatic amputation (Figures 3A, 3B).

Laceration of size 15 cm x 12 cm was present over the front of the right leg in mid-part, along with an open comminuted fracture of the tibia (Figure 3C). The patient was shifted to the emergency operation theatre, where debridement of bilateral forearm stump was done along with external fixation of fracture of the right tibia and k-wire fixation of the right olecranon. Ear examination revealed bilateral traumatic subtotal perforation of the tympanic membrane, for which tympanoplasty was advised. On the fifth day, the patient developed fever. A swab from the left forearm showed the presence of Pseudomonas aeruginosa, and a swab from the right leg showed the presence of Citrobacter koseri, for which appropriate antibiotics were administered. On the sixth day, re-debridement with stump was done over both forearms, along with debridement and vacuum-assisted closure of the right leg. On the 14th day, re-debridement and anterolateral thigh flap cover of the right leg was done. However, the condition of the right leg wound deteriorated, and below-knee amputation was performed on the 27th day. The patient was discharged on the 35th day with follow-up advice.

## Discussion

A chemical explosion may cause multiple injuries varying from minor burns to life-threatening amputations, penetrating injuries and consequent death. The leading component behind these injuries is the formation of a massive amount of kinetic energy over a short period in the form of the blast wave [1]. The explosives are divided into two types on the basis of their production source: Manufactured and Improvised. Manufactured explosives are often used by armed forces and are rigorously subjected to quality control measures. In comparison, improvised explosives are usually produced in small quantities using a weapon or chemical outside of its designated purpose [1,4]. Accidental explosions could occur while making or using improvised explosive devices carelessly, as seen in the reported cases.

The chemical substance viz. potash (pot-ash) originates from an early manufacturing process where potassium was excreted from charcoal and purified by evaporating the leachate in enormous iron vessels [5]. The term potash has been commonly used to describe the fertilizer forms of potassium. Both the chemicals (sulfur and potash) are used as fertilizers in farming and are easily available in the market at very low prices all over India. Manufacturing, possessing, and importing any explosive made up of or containing sulfur in admixture with chlorate of potassium or any other chlorate except for scientific purposes, production of heads of matches in the matchbox, paper caps for toy pistols, and percussion caps in railway fog signal is prohibited under Indian Explosives Act, 1884 [6]. Yet, people use them to make indigenous firecrackers to celebrate marriages and festivals, and as explosive bombs to spread terror or harm someone [3].

The type of potash used in the reported case was potassium chlorate. It has strong oxidizing properties [7]. On the other hand, sulfur has inherent flammable properties and is very sensitive to friction [8]. Sulfur can be ignited by a spark or friction and produce heat, triggering the decomposition of potassium chlorate. The oxygen released by the potassium chlorate can further aggravate the burning of sulfur [7]. The result is sound production, for which the farmers use this mixture to scare away the animals from damaging their crops. A small quantity of the mixture is sufficient to produce much noise. In the reported cases, the victims were unaware of the chemical’s explosive potential and were grinding the solid forms of both explosive compounds together rather than separately. Another possible mechanism regarding the chemical reaction in the reported case is the reaction between sulfuric acid and potassium chlorate [9]. The sulfur powder available in the market contains various amounts of sulfuric acid. Potassium chlorate reacts with the sulfuric acid and forms potassium sulfate (2 KClO_3_ + H_2_SO_4_ →2 HClO_^3^_ + K_2_SO_4_), a highly reactive explosive [9].

The pattern of injuries following an explosion depends on the composition and quantity of the materials, the neighboring environment, the range between the injured party and the blast, and any environmental risks or intervening physical barriers [1,4]. The injuries produced by explosives can be classified into four groups: Primary, secondary, tertiary, and quaternary [1,4]. Primary injuries are produced due to over or under-pressurized blast waves, affecting mainly gas-filled organs. Secondary injuries are produced from flying debris and bomb fragments. They are the most common cause of death and mortality in an explosion event. Most of the secondary injuries are penetrating and blunt trauma to the body resulting in fractures, traumatic amputation, and soft tissue injuries. Tertiary injuries are produced when the victim is thrown away by blast wind sustaining blunt and penetrating injuries. Quaternary injuries are any injury, disease, or complication due to an explosion that is not included in the aforementioned types including burns, inhalational injuries, toxic effects, and crush injuries [1,4]. Depending upon the safety distance categories of Explosive rules of the Explosive Act of India, the reported cases come under the category X, i.e., those explosives, which have a fire or a slight explosion risk or both but the effect of which will be local [10]. The violent impact of the flying missiles during the explosion caused multiple abrasions, bruises, and punctate lacerations called a Marshall’s triad, which is evident in the reported cases [11]. Traumatic amputations of the limbs and rupture of the tympanic membrane were due to the interaction between pressure waves and body parts in close contact and without an intervening object. These injuries can be labeled as primary blast injuries. Rupture of the tympanic membrane is the most common primary organ injury in explosions [1]. The pressure required to cause damage to the air-filled organs (56 to 76 pounds per square inch) is much higher than the pressure required to cause rupture of the tympanic membrane (around 5 pounds per square inch) [1]. Thus, primary explosion injuries to other air-containing organs are less likely if the tympanic membrane is not ruptured, as evident in both victims of case 1. Irregular skin burns and traumatic tattooing are typical wound patterns of firework devices [2]. In the reported cases, the type of explosives chemicals was low order firework type, so the victim suffered superficial burns over various body parts along with powder tattooing due to indentation of the unburned chemical mixture into the layers of the skin. Traumatic tattooing and punctate contusions and lacerations can be labeled as secondary blast injuries. The traumatic amputations of two of the victims' limbs were due to their proximity to the point of explosion. As in case 1, the younger brother stood behind the elder brother; he was solely subjected to heat effect and blast tattooing.

Giri et al. reported an autopsy case of fatality due to an accidental blast while mixing chemicals for an improvised animal scaring gun, in which the victim used the same chemical mixture which was used in the reported cases [3]. They reported yellowish powdery stains over the body and superficial to deep burns over the various parts of the front of the body. The victim suffered traumatic amputation of the little finger and fifth metatarsal of the left hand due to closeness to the point of explosion. Bony defect of size 5cm x 2cm was present over frontal bone. The death was due to a laceration of the frontal lobe of the brain along with subdural and subarachnoid hemorrhage. Nagpal et al. reported a case of ocular blast injury due to an accidental blast while mixing sulfur and potash [9]. The victim suffered multiple charred wounds on the face with numerous yellowish-white granular foreign body particles planted into the layers of the cornea. Due to the small quantity of mixture, the victim suffered only minor facial and ocular injuries.

## Conclusions

These cases highlight spectra of explosion injuries by low-order explosives (sulfur and potash) ranging from extensive tattooing to traumatic amputations of the limbs, based on proximity to the point of explosion. These incidents are consequences of underestimation of the explosive potential and dangers of using sulfur and potash. Injudicious and reckless use of such chemicals can lead to significant morbidity and mortality. Hence, increasing public awareness and stringent legislation pertaining to its availability and use is warranted.
